# Rhodoliths holobionts in a changing ocean: host-microbes interactions mediate coralline algae resilience under ocean acidification

**DOI:** 10.1186/s12864-018-5064-4

**Published:** 2018-09-24

**Authors:** Giselle S. Cavalcanti, Priya Shukla, Megan Morris, Bárbara Ribeiro, Mariah Foley, Michael P. Doane, Cristiane C. Thompson, Matthew S. Edwards, Elizabeth A. Dinsdale, Fabiano L. Thompson

**Affiliations:** 1 Biology Institute, Federal University of Rio de Janeiro (UFRJ), Rio de Janeiro, RJ 21941-599 Brazil; 2 Department of Biology, San Diego State University, San Diego, CA 92182 USA

**Keywords:** Climate change, Coralline crustose algae, Host-microbe interactions, Metagenomics, Ocean acidification, Rhodolith

## Abstract

**Background:**

Life in the ocean will increasingly have to contend with a complex matrix of concurrent shifts in environmental properties that impact their physiology and control their life histories. Rhodoliths are coralline red algae (*Corallinales, Rhodophyta*) that are photosynthesizers, calcifiers, and ecosystem engineers and therefore represent important targets for ocean acidification (OA) research. Here, we exposed live rhodoliths to near-future OA conditions to investigate responses in their photosynthetic capacity, calcium carbonate production, and associated microbiome using carbon uptake, decalcification assays, and whole genome shotgun sequencing metagenomic analysis, respectively. The results from our live rhodolith assays were compared to similar manipulations on dead rhodolith (calcareous skeleton) biofilms and water column microbial communities, thereby enabling the assessment of host-microbiome interaction under climate-driven environmental perturbations.

**Results:**

Under high *p*CO_2_ conditions, live rhodoliths exhibited positive physiological responses, i.e. increased photosynthetic activity, and no calcium carbonate biomass loss over time. Further, whereas the microbiome associated with live rhodoliths remained stable and resembled a healthy holobiont, the microbial community associated with the water column changed after exposure to elevated *p*CO_2_.

**Conclusions:**

Our results suggest that a tightly regulated microbial-host interaction, as evidenced by the stability of the rhodolith microbiome recorded here under OA-like conditions, is important for host resilience to environmental stress. This study extends the scarce comprehension of microbes associated with rhodolith beds and their reaction to increased *p*CO_2_, providing a more comprehensive approach to OA studies by assessing the host holobiont.

**Electronic supplementary material:**

The online version of this article (10.1186/s12864-018-5064-4) contains supplementary material, which is available to authorized users.

## Background

Changes in ocean carbonate chemistry driven by increasing anthropogenic carbon dioxide (CO_2_) emissions promote ocean acidification (OA). Increasing ocean CO_2_ uptake results in the elevation of partial pressure of CO_2_ (*p*CO_2_), lower pH levels, and lower carbonate saturation of seawater, all of which control the calcification process [1, 2]. The OA-induced changes in seawater carbonate chemistry may affect organisms that accrete carbonate as part of their physical structure since precipitating CaCO_3_ would become less efficient [3–6]. Therefore, OA poses a serious threat to calcifying marine organisms (e.g. corals, coralline algae, mollusks, echinoderms) by affecting their growth and reproduction [7–10]. Coralline algae are considered particularly sensitive to OA since they precipitate high Mg-calcite carbonate skeletons, the most soluble form of CaCO_3_ [11], which constitute more than 80% of the dry mass of the thallus [12].

As both photosynthesizers and calcifiers, coralline algae may respond in multiple ways to ocean acidification. Calcification rates in coralline algae are thought to be directly related to their photosynthetic rates, but it is still not clear how a high-CO_2_ environment might affect this group of photosynthetic calcifying algae [13]. Elevated CO_2_ levels might impact calcifying algae by impairing biomineralization, due to decreased seawater carbonate (CO_3_^2−^) availability as pH falls; but it also may promote photosynthesis (within tolerance limits), as the availability of bicarbonate (HCO_3_^−^) increases [9]. Indeed, the expected parabolic relationship between declining pH and coralline algal fitness may explain the varied responses to declining pH and elevated *p*CO_2_ that have been recorded to date [14–23]. To ascertain the extent till which these opposing effects offset each other represents a crucial step in understanding the potential overall impact of OA on crustose coralline algae.

Among coralline algae (*Corallinales, Rhodophyta*), rhodoliths are the free-living non-geniculate species, which form extensive beds worldwide by accumulating live and dead thalli [24–26]. Rhodolith beds provide a multidimensional structure for organisms to settle, thus increasing biodiversity and acting as a nursery ground [27]. In addition, rhodolith beds produce calcium carbonate and dissolved organic carbon, which support the proliferation of other trophic levels within the ecosystem, including microbes [28, 29]. The microbes associated with the rhodolith algae are comprised of a diverse assemblage of bacteria, small eukaryotes, and viruses. Collectively, the algae and associated microbes function as an ecological unit termed the holobiont [30, 31]. Rhodoliths support a stable microbiome that maintains an extremely homogenous composition even in specimens that occur 100 km from each other [28]. However, whether the rhodolith microbiome remains stable under environmental perturbations, such as stressors from ocean acidification, is unknown.

Recent work has focused on the effects of OA and co-occurring environmental changes on coralline algae (e.g. reviewed [13]). However, studies assessing the response of coralline algae-associated microbial communities remain incipient despite the acknowledged role of bacterial communities in algal biology [30, 32–34]. Furthermore, the comprehension of algal-bacterial interactions are key to elucidate processes important for the acclimation of the holobiont to environmental changes [35–38]. For example, changes in the microbial community associated with crustose coralline algae (CCA) and a concomitant reduction in coral larval settlement under OA conditions were previously documented [39]. A second study assessing the microbial communities of calcifying reef taxa (i.e. corals, CCA, foraminifera, sea urchins) found their microbiome remained stable under experimental exposure to reduced pH seawater but CCA and foraminifera were sensitive to the combined effects of ocean warming and acidification [40]. Further studies should elucidate the mechanisms through which microbial shifts affect host health and fitness. Nevertheless, the sensitivity of microbial taxa associated with CCA and foraminifera highlight the need for a holobiont centric-approach to assess vulnerability to climate change.

Here, we report the first metagenomic analysis of live rhodoliths, the microbial biofilm that developed on the calcareous skeleton or thalli that was devoid of tissue (subsequently referred to as dead rhodolith), and the surrounding water column exposed to near future OA conditions. We aimed to investigate the influence of increased *p*CO_2_ on the physiological and microbiome responses of rhodolith-forming crustose coralline algae under controlled experimental mesocosms. Specifically, rhodolith physiology was measured through photosynthesis and calcification assays, and the stability of its associated microbiome was characterized using whole-genome shotgun metagenomic sequencing analysis. Responses in the live rhodolith microbiomes were compared against those of the dead rhodoliths and water column controls to identify the potential for host-mediated influence on microbiome resilience. We hypothesized that, due to the essential roles that microbiomes play in normal physiological host function and susceptibility to environmental stress, the live rhodoliths would exhibit a stable microbiome under high *p*CO_2_ exposure while the algal physiology was not impaired. Our results support the hypothesis that coralline algae rhodoliths harbor a tightly controlled and stable microbiome, important to the holobiont resilience in face of imminent ecological stressors.

## Results

### Rhodolith photosynthesis and calcification

**Fig. 1:**
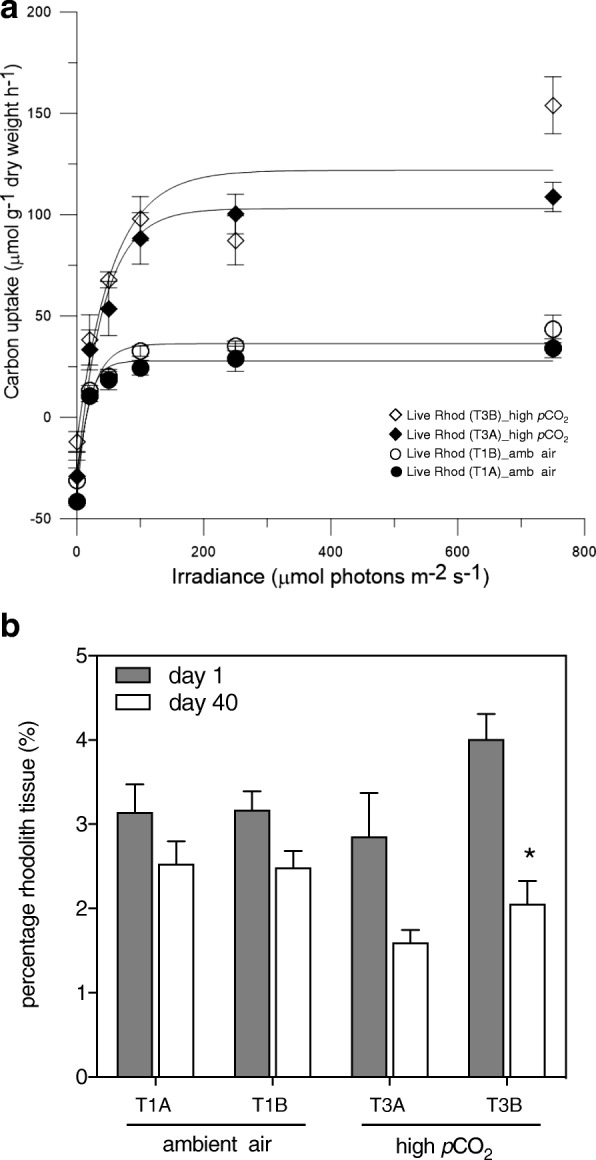
Rhodolith physiology was not impaired under elevated *p*CO_2_. **a** Photosynthesis, measured as carbon uptake by live rhodolith, under ambient air (tanks 1A and 1B) and high CO_2_ (Tanks 3A and 3B), at the end of the 40-day experiment. Rhodolith specimens (*n* = 3 per mesocosm) were cultured under 6 light levels (0, 25, 50, 100, 250, and 750 μmols photons m^− 2^ s^− 1^) for one hour each (in triplicate). Best-fit curves were calculated for each treatment and photosynthetic maximum (P_max_) and photosynthetic efficiency (α) were calculated from these using Platt et al. (1975). **b** Proportion of live tissue in rhodolith holobionts under ambient air (tanks 1A and 1B) and high CO_2_ (Tanks 3A and 3B), measured through decalcification assay, at the beginning (day 1) and ending (day 40) of the experiment. (*) denotes statistically significant difference over time

Photosynthetic carbon uptake by live rhodoliths differed between *p*CO_2_ treatments at the end of the experiment (Fig. 1a). Live rhodoliths under high *p*CO_2_ had higher maximum potential photosynthetic rates (P_max_), two-fold higher than observed in algae under ambient air. In contrast, the photosynthetic efficiency (α) was not affected by *p*CO_2_ treatment.

Rhodolith calcification was not significantly impaired during the experiment. The amount of calcium carbonate was calculated through the decalcification assay and revealed that, over time, live rhodoliths lost tissue, not skeleton (Fig. 1b). The proportion of live tissue (percentage of organic material) on rhodoliths under ambient air and, more markedly, under high CO_2_ decreased from the start of the experiment (day 1) to the end (day 40). Nevertheless, only one high CO_2_ mesocosm displayed a statistically significant decline over time (*p* = 0.008). The percentage of inorganic matter (skeleton) increased 0.65% for the rhodoliths under ambient air treatment (from 96.85 and 96.82% at day 1, to 97.47% and 97.51% at day 40 for T1A and T1B, respectively) and increased 1.66% for the rhodoliths under high CO_2_ treatment (from 97.15 and 95.99% at day 1, to 98.41% and 97.94% at day 40 for T3A and T3B, respectively). Rhodoliths showed a higher percentage of inorganic carbon (calcareous skeleton) in the high *p*CO_2_ treatment.

### Rhodolith microbiome

Whole genome sequencing of total DNA extracted from the live rhodolith holobionts, the dead rhodolith biofilm, and the seawater from the holding tanks yielded 28 metagenomic libraries totaling 16.31 million quality reads, with an average of 108,476,267 bp per metagenome (Additional file 1). The sequenced reads were annotated using the MG-RAST [41]. Subsequent comparison with the NR database from GenBank provided taxonomic annotations for 17% to 49% of rhodolith metagenomes and 23% to 48% of seawater metagenomes. The function assignments obtained from the SEED database provided metabolic annotations for 19% to 63% of rhodolith metagenomes and 29% to 59% of seawater metagenomes. Bacteria were the most abundant domain in all metagenomes, with higher average proportions in both seawater (98.7%) and dead rhodoliths microbiomes (96.6%) compared with the live rhodolith holobionts (73.2%). The Eukaryota and Archaea domains comprised on average 25.0% and 1.4%, respectively, of live rhodolith metagenomes. Eukaryotes represented 3.0% of dead rhodolith metagenomes and 0.6% of seawater metagenomes, while Archaeans comprised only 0.2% of the sequences from seawater and dead rhodoliths biofilm. Taxonomic similarities in Archaea families and Eukarya classes revealed a stable community composition in the live rhodolith metagenomes (Additional file 2).

**Fig. 2:**
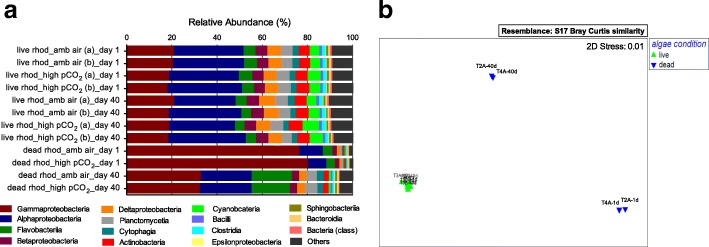
Rhodolith microbiome was stable under elevated *p*CO_2_. **a** Relative abundance of the 15 most abundant bacterial classes in dead (calcareous skeleton biofilm) and live rhodolith metagenomes, under ambient air and high CO_2_ treatments. **b** MDS plot for the relative abundance of rhodolith bacterial taxonomic similarities by class contribution (Bray Curtis similarity). Algae condition was the grouping variable, represented by green triangle for live rhodolith (T1A, T1B - live rhod_amb air; T3A, T3B - live rhod_high CO_2_) with no effect from time (1 day versus 40 days) or *p*CO_2_ level (ambient air versus high CO_2_) variables; and blue triangle for dead rhodolith (T2A - dead rhod_ high CO_2_; T4A - dead rhod_amb air), at the beginning (day 1) and ending (day 40) of the experiment

The bacterial taxonomy of the live rhodolith microbiomes was stable across time regardless of *p*CO_2_ treatments or sampling period (Fig. 2), which can be visualized as all 8 metagenomes taken from the live rhodoliths clustered together on the nMDS plot (Fig. 2b). In comparison, the dead rhodolith biofilm metagenomes displayed a shift during the course of the experiment. The shift in the dead rhodolith microbial community observed during the experiment resembled a biofilm succession, where the Gammaproteobacteria, predominant in day 1, were partially replaced, by Alphaproteobacteria and Flavobacteria towards the end of the experiment (day 40) (Fig. 2a). Over the 40 days experiment, the microbiome that formed on the dead rhodoliths showed a decrease in the relative abundance of gram-negative marine copiotrophic Gammaproteobacteria families *Pseudoalteromonadaceae* (− 84.23%), *Alteramonadaceae* (− 69.13%), *Colwelliaceae* (− 89.66%), *Pseudomonadaceae* (− 41.32%), *Shewanellaceae* (− 66.76%), *Vibrionaceae* (− 55.50%), *Oceanospirillaceae* (− 64.02%). After the 40 days exposure to high *p*CO_2_, an increase was observed in the relative abundance of the Alphaproteobacteria families *Rhodobacteraceae* (+ 117.41%), *Hyphomonadaceae* (+ 261.95%), *Phyllobacteriaceae* (+ 372.82%), *Rhizobiaceae* (+ 311.97%), *Rhodospirillacecae* (+ 249.17%) as well as in the Cytophaga-Flavobacterium-Bacteroides (CFB) families *Cytophagaceae* (+ 327.82%), *Flavobacteriaceae* (+ 334.03%), *Bacteroidaceae* (+ 249.17%) (Additional file 3).

**Fig. 3:**
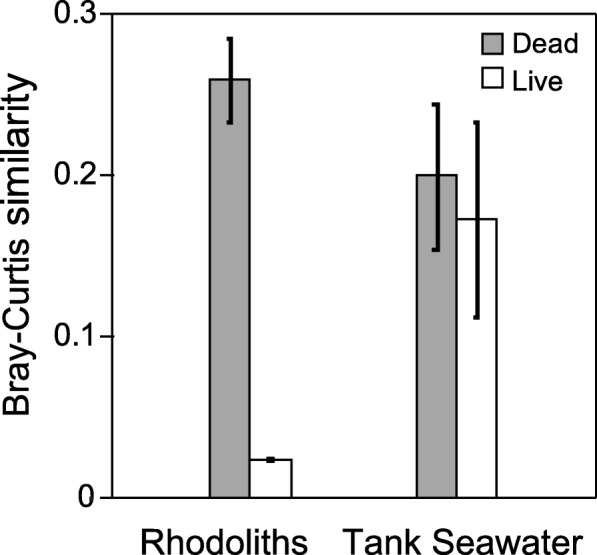
Changes in bacterial communities over time. Bray-Curtis similarities in Bacterial class composition between the beginning (day 1) and end (day 40) of the experiment, for live and dead incubations, and rhodolith thalli vesus tank seawater samples. Bacterial communities associated with live rhodoliths were similar over the course of the experiment, whereas significant changes in community structure were observed with the dead rhodoliths biofilms and both seawater column treatments

The live rhodoliths showed a stable microbiome that did not change with *p*CO_2_ levels or time (Fig. 2). To compare the amount of change in bacterial taxonomy that occurred over time, we calculated the mean change in Bray-Curtis similarity among each of the rhodolith thallus treatments (i.e. live and dead rhodoliths), and each of the two seawater locations (i.e. water column over the live and dead rhodoliths) (Fig. 3). Changes in bacterial communities between day 1 and day 40 were statistically significant between live and dead treatments (2-way ANOVA, *p* = 3.05 × 10^− 3^), but not significant between location (tank seawater versus rhodolith thalli) (2-way ANOVA, *p* = 1.07 × 10^− 1^). However, the algal treatment factor (live versus dead) interacted with location (tank seawater versus rhodoliths thalli) (2-way ANOVA, *p* = 8.12 × 10^− 3^). Thus, the larger variation (statistically significant) in bacterial communities observed in the seawater holding tank and dead rhodolith biofilm throughout the experiment was mainly driven by the comparison with the stable microbiome observed in live rhodoliths (Fig. 3). Tukey’s post hoc tests revealed that temporal changes in the bacterial communities associated with the live rhodoliths were significantly different from those associated with the dead rhodoliths (*p* = 5.34 × 10^− 3^), as well as with the water column over both the dead rhodoliths (*p* = 1.55 × 10^− 2^), and live rhodoliths (*p* = 2.76 × 10^− 2^). No temporal differences were observed among pairs of the other three treatments. Thus, the bacterial communities associated with live rhodoliths were similar over the course of the experiment, whereas significant changes in community structure were observed with the dead rhodoliths and both seawater column treatments (Fig. 3).

Multiple group analysis comparing rhodolith microbiome between algae condition (live versus dead) and *p*CO_2_ levels identified Cyanobacteria (*p* = 3.81 × 10^− 6^), Actinobacteria (*p* = 3.79 × 10^− 3^), Deltaproteobacteria (*p* = 1.30 × 10^− 2^), Betaproteobacteria (*p* = 1.62 × 10^− 2^), Clostridia (*p* = 1.77 × 10^− 2^), and Gammaproteobacteria (*p* = 2.12 × 10^− 2^) as bacterial classes with average abundance above 1.0% that were significantly different (ANOVA *p*-value < 0.05).

### Microbial diversity in the seawater holding tanks

**Fig. 4:**
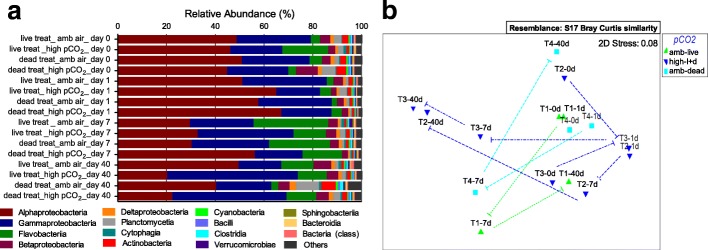
Water column microbial communities shifted under elevated *p*CO_2_. **a** Relative abundance of the 15 most abundant bacteria classes in seawater metagenomes. **b** MDS plot for the relative abundance of the seawater holding tanks taxonomic similarities by class contribution (Bray-Curtis similarity). Seawater metagenomes were represented by *p*CO_2_ treatment, green triangle for live rhodolith in ambient air (T1 – tank 1), blue triangle for high *p*CO_2_ treatments - both calcareous skeleton (T2 – tank 2) and live rhodolith (T3 – tank 3), light blue square for dead rhodolith (calcareous skeleton in ambient air (T4 – tank 4). The respective color dashed lines connect the temporal sequence of each specific *p*CO_2_ treatment in the plot. Water metagenomes from each one of the four tanks were sequenced from days 0, 1, 7, 40 (0d, 1d, 7d, 40d, respectively)

Microbial diversity within seawater holding tanks varied through time and was affected by *p*CO_2_, regardless of the presence or absence of live rhodoliths (Fig. 4). Alphaproteobacteria (43.92%), Gammaproteobacteria (28.71%), Flavobacteria (10.49%), Betaproteobacteria (4.10%), Planctomycetia (2.38%), Actinobacteria (1.85%), Deltaproteobacteria (1.33%) were the classes with average abundance above 1.0% that contributed to this variation (Fig. 4a). When analyzing seawater metagenomes, the rhodolith condition (live versus dead) was a poor predictor of microbial community structure. In contrast, an effect of the different *p*CO_2_ treatments was observed, where holding tanks kept under high CO_2_ were closely related in the MDS plot (Fig. 4b). The microbial community within the high *p*CO_2_ holding tanks from both live and dead rhodoliths (dark blue triangles in Fig. 4b) showed the same trajectory on the MDS plot. In particular, the Alphaproteobacteria contribution decreased from over 60% of the community on day 1 to about 20% of the community on day 40 in both water columns high *p*CO_2_ treatments (Fig. 4a). Taxonomic differences among the water profiles across the experiment (time grouping variable) were driven by the classes Flavobacteria (*p* = 8.82 × 10^− 3^), Deltaproteobacteria (*p* = 1.06 × 10^− 2^), and Alphaproteobacteria (*p* = 1.87 × 10^− 2^) (ANOVA *p*-value < 0.05).

### Functional profile of rhodoliths and seawater

**Fig. 5:**
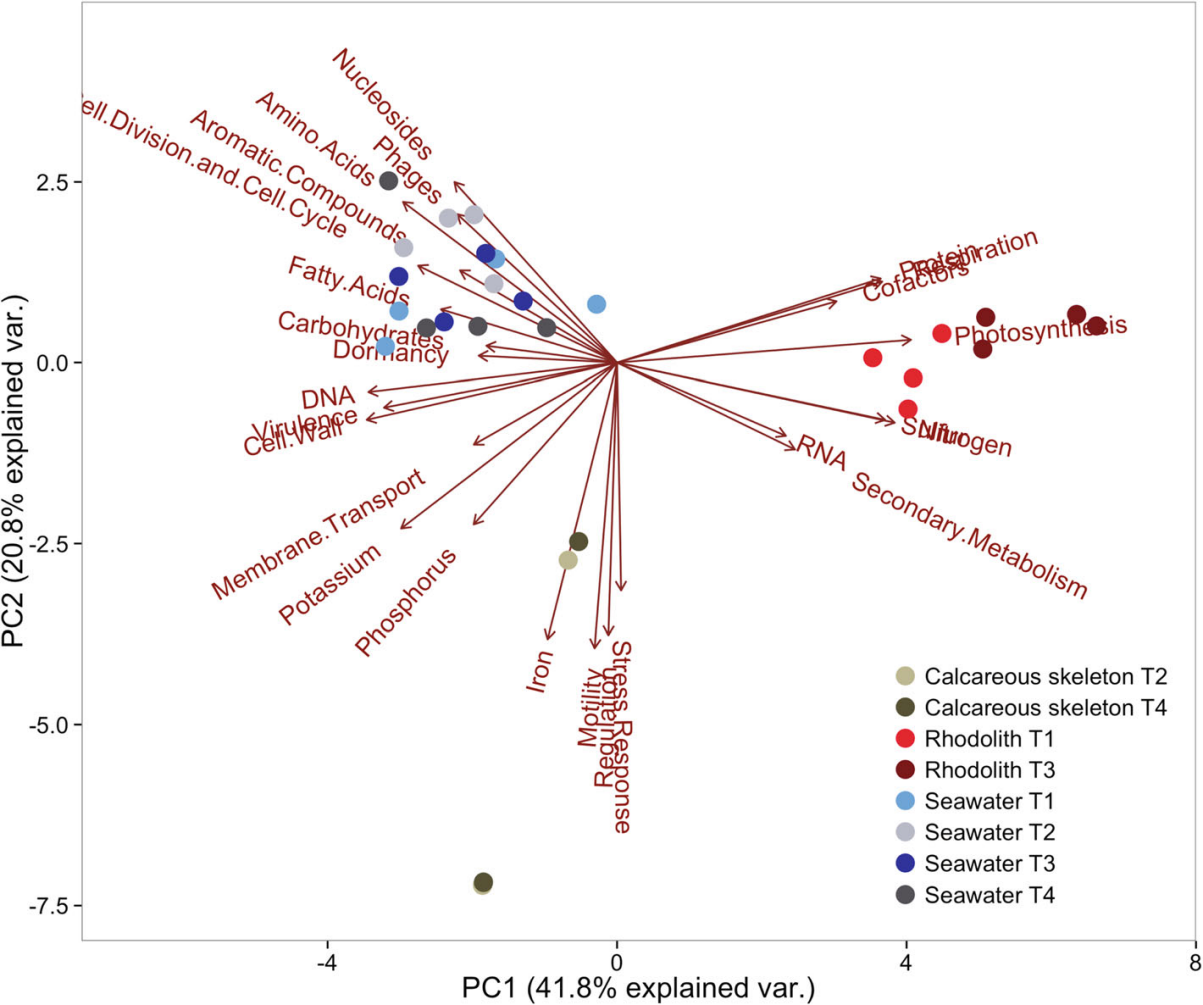
Functional analysis grouped the microbial communities by live rhodolith, dead rhodolith, or seawater. Principal component analysis (PCA) depicting the correlations between predictor variables (SEED database level 1 subsystems - red arrows) and the metabolic similarity in rhodolith (live and dead) and seawater metagenomes for all treatments (28 metagenomes) during the experiment. The length of the arrow represents how well the parameter explains the distribution of the data. PCA grouping live rhodolith microbiome under ambient air (red circle; T1 – Tank 1) and high CO_2_ (dark red circle; T3 – Tank 3); dead rhodolith biofilm under ambient air (beige circle; T4 – Tank 4) and high CO_2_ (brown circle; T2 – Tank 2); and seawater metagenomes under four combined treatments: live algae, ambient air (light blue circle; seawater T1 - Tank 1), live algae, high CO_2_ (blue circle; seawater T3 - Tank 3), dead rhodolith, ambient air (gray circle; seawater T4 - Tank 4), dead rhodolith, high CO_2_ (dark gray; seawater T2 - Tank 2) by functional contribution in first level of hierarchy (level 1)

Predicted metabolic similarities in rhodolith and water metagenomes under combined treatments (algae condition, *p*CO_2_, time) revealed three clusters in the PCA plot: live rhodoliths, biofilm on dead rhodoliths, and holding tank water (Fig. 5). No statistically significant differences were found between *p*CO_2_ levels within both dead and live treatments for either rhodolith holobionts or seawater column (two-group comparisons) (Additional file 4).

The dead rhodolith microbiome displayed higher mean relative frequencies assigned to metabolic biofilm formation when compared to seawater and live rhodoliths, including carbohydrates [*aminosugars* (*p* = 2.57 × 10^− 6^), *polysaccharides* (*p* = 5.25 × 10^− 6^), *di- and oligosaccharides* (*p* = 3.70 × 10^− 5^), *organic acids* (*p* = 2.55 × 10^− 2^)]; cell wall and capsule (*p* = 4.03 × 10^− 6^); iron acquisition and metabolism (*p* = 4.24 × 10^− 2^); membrane transport [*protein secretion system, Type III* (*p* = 5.45 × 10^− 3^), *membrane transport* (*p* = 1.36 × 10^− 2^)], motility and chemotaxis [*flagellar motility* (*p* = 1.07 × 10^− 3^), *motility and chemotaxis* (*p* = 2.56 × 10^− 3^)] and stress response (*p* = 4.60 × 10^− 2^).

In comparison with dead rhodolith and seawater, live rhodolith metagenomes under both ambient air and high CO_2_ conditions showed increased proportion of sequences associated with the metabolism of nitrogen (*p* = 1.26 × 10^− 6^), protein [*protein biosynthesis* (*p* = 3.89 × 10^− 3^), *protein folding* (*p* = 9.11 × 10^− 3^)], RNA [*transcription* (*p* = 2.50 × 10^− 8^), *RNA metabolism* (*p* = 7.07 × 10^− 8^)] and sulfur [*inorganic sulfur assimilation* (*p* = 1.21 × 10^− 8^), *organic sulfur assimilation* (*p* = 1.20 × 10^− 4^)], as well as in the photosynthesis [*electron transport and photophosphorylation* (*p* = 7.16 × 10^− 19^), *light-harvesting complexes* (*p* = 1.76 × 10^− 13^)] and respiration [*respiration* (*p* = 4.95 × 10^− 7^), *ATP synthases* (*p* = 1.78 × 10^− 5^), *electron donating reactions* (*p* = 2.29 × 10^− 2^)].

## Discussion

The results of this experimental study showed that the microbial community structure in the seawater and the dead rhodoliths biofilm changed during the course of the experiment, while the live rhodolith microbiome remained stable. Further, the algae photosynthetic capacity increased after exposure to high *p*CO_2_. The inorganic carbon rates (percentage of calcareous skeleton) were higher in rhodoliths under high *p*CO_2_ treatment, but organic content (live tissue) was lower, suggesting that the excess energy from photosynthesis went towards deposition of CaCO_3_ rather than growth.

### Enhanced photosynthetic performance offsets rhodolith calcification under enriched CO_2_

Consistently elevated rates of carbon uptake during photosynthesis were observed in rhodoliths under high *p*CO_2_, as indicated by a higher P_max_, compared with observations for rhodoliths under ambient air treatment. Photosynthetic efficiency (α) was unaltered regardless of *p*CO_2_. A significant increase in sequences associated with photosynthetic activity was observed among live rhodolith metagenomes compared to water column and dead rhodolith biofilm. Accordingly, the proportion of sequences assigned to photosynthesis metabolism was 24.31% higher under elevated CO_2_ compared to the ambient air live rhodolith metagenomes. Photosynthesis draws carbon (CO_2_ or bicarbonate) from total C_I_ [42], whereas calcification takes up inorganic carbon and releases CO_2_. Calcification is an energy-demanding process and photosynthesis can provide the energy needed to support the precipitation of carbonate skeletons. In our study, the inorganic carbon rates (percentage of calcareous skeleton) were higher in the rhodolith under high *p*CO_2_ treatment compared to rhodolith under ambient air treatment, but organic content was lower. Therefore, it is possible that more inorganic carbon was allocated to deposition of CaCO_3_, but at the cost of growth.

The research on OA in mesocosms poses challenges due to the constraints in fully reproducing field conditions in the laboratory. Coralline algae have shown strong species-specific responses to elevated *p*CO_2_, where two closely related species in the same genus ﻿presented disparate responses [43], or variable responses could be recorded between two studies on the same species [44]. However, the variable responses of coralline algae could be driven by the timescale of the physiological measurements, and the acclimation period or the time of the year experiments were done [45]. Our results and the evidence from short-term studies confirm the stimulation of photosynthesis in coralline algae with increased CO_2_ availability.

Photosynthesis rates and the surrounding concentration of inorganic carbon are related to calcification rates in coralline algae. Similarly, calcification can stimulate algal photosynthesis, if it acts as a carbon concentration mechanism, as proposed previously [46]. Briefly, according to the “trans calcification” mechanism, an external carbonic anhydrase converts bicarbonate ions to CO_2_ for photosynthesis, which, in turn, releases the carbonate (CO_3_^2−^) used in algal calcification [46]. Photosynthetic activity in macroalgae leads to a pH increase in the intercellular spaces along in the diffusion boundary layer. The persistent diffusion boundary layer at the surface of coralline algae creates a pH microenvironment very different from the mainstream seawater [47, 48] and the stability in the microbiome with increasing *p*CO_2_ confirms the control the algae exert on the boundary layer. As rhodoliths precipitate CaCO_3_ in their cell walls, the increase in pH shifts the equilibrium toward an increase in CO_3_^2−^ concentration [49, 50], which promotes precipitation of CaCO_3_. Thus, the positive effect of photosynthesis on calcification to a certain extent could offset CaCO_3_ dissolution in calcifying algae in response to increased *p*CO_2_ [49–51]. In the rhodoliths assessed in our study, the increase in photosynthesis may have supported increased calcium carbonate production.

Previous studies have demonstrated a parabolic response in coralline algae physiology to pH and *p*CO_2_ [14, 18, 23]. Yet, most long-term experimental studies show a decrease in calcification and enhanced dissolution in calcifying species under elevated CO_2_ concentrations [7, 9, 22, 44, 52]. Additionally, as the thermal stress is known to exacerbate the adverse effect of OA on algal physiology [15, 35, 44, 53, 54], in the climate-change scenario, where CO_2_ and temperatures are elevated, the photosynthesis-calcification metabolic processes in calcifying macroalgae might become uncoupled [49]. However, more complete mechanistic understanding of the complex metabolic links between inorganic carbon uptake pathways, photosynthesis, and calcification in coralline algae is still necessary to better predict the physiological limits of these important autotrophs to climate change stressors.

### Rhodolith microbiome stability is crucial to holobiont resilience

Rhodolith function is intimately linked to the composition and structure of their associated microbiome. Due to their diverse metabolic traits, microorganisms play important roles in macroalgae physiology, as suggested for instance, in the biomineralization process [28]. When considering the phenotypic plasticity of coralline algae to changing environments, the microbiome offers significant but often underappreciated potential. Microbial symbionts can promptly respond to changing environmental conditions, offering a powerful mechanism for acclimatization [55]. However, instabilities in host-microbe interactions can have significant functional consequences for the holobiont physiology and susceptibility to environmental stressors. Healthy hosts normally possess relatively stable microbiomes, where the host-microbes normal regulation leads to beneficial microbiome configurations [56]. Here, we observed a tightly controlled and stable rhodolith microbiome during elevated *p*CO_2_ conditions, resembling a healthy holobiont.

**Fig. 6:**
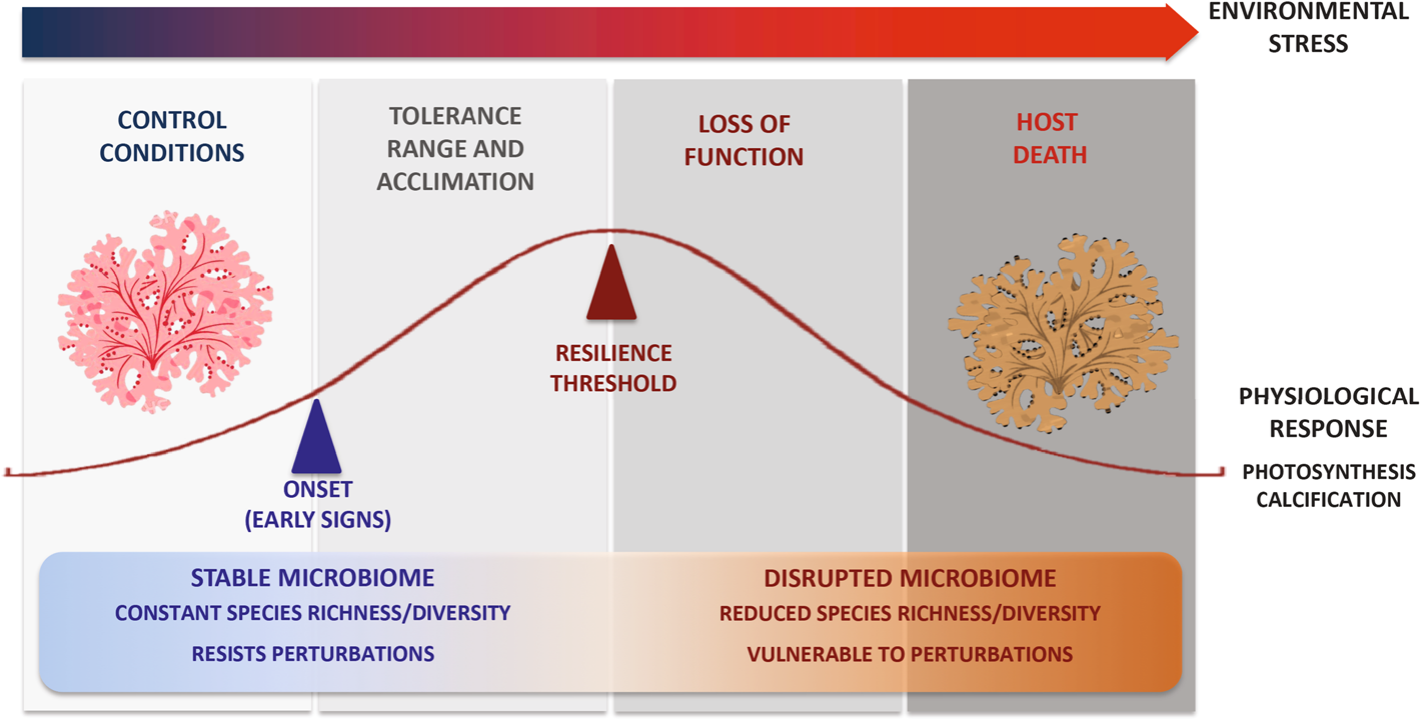
Conceptual overview of the expected parabolic relationship between climate change stressors and rhodolith holobiont fitness. Under normal conditions, healthy rhodoliths possess stable microbiomes, important to the holobiont function. However, beyond thresholds of algal physiological tolerance to climate change stressors, the disruption of positive host-microbiome interactions occurs, detrimentally affecting the holobiont fitness

Nevertheless, our experimental design assessed the rhodolith holobiont response to a single predicted climate change stressor. It is possible that the single stress applied here was within the algae physiologic tolerance limits, i.e. below the organismal vertices curve response, and therefore was not sufficient to cause dysbiosis. We hypothesize that the unbalanced tradeoff between photosynthesis and calcification in coralline algae under environmental stress occurs concomitantly with the disturbance of the algae microbiome. For example, combined stressors such as high temperature and high *p*CO_2_ could impair host physiology disrupting beneficial host-microbiome interactions (Fig. 6).

Metagenomic analysis provides structural and putative functional information of the host-microbial interactions under study. Future studies using metatranscriptomic analyses that focus on gene expression of active members of microbial communities might reveal microbial responses to rapid environmental change, thereby linking structural shifts to community function [57]. Still, valid predictions of coralline algae rhodolith response to ocean change rely on bridging gaps between diverse omics approaches and earth system science. The development of an improved understanding of coralline algae ecophysiology, considering the interactions between algae and associated bacteria, is key to assess environmental effects on the holobiont. Our study provides a relevant step in this direction.

## Conclusions

OA poses an ever-increasing threat to the ecologic balance and stability of rhodolith bed systems via differential impacts on coralline algae physiology. Rhodolith widespread distribution hints at the resilience of this algal group that remained chief components of benthic marine communities through considerable environment fluctuations (i.e. temperature, light) over geologic times [58]. Whole genome shotgun sequencing performed here on a variety of rhodolith bed constituents revealed a stable live rhodolith microbiome still thriving under elevated *p*CO_2_ conditions. In contrast, microbial shifts in the seawater column and in the coralline skeleton biofilm were observed. These findings reinforce the existence of a close host-microbe functional entity, where the metabolic crosstalk within the rhodolith as a holobiont could be exerting reciprocal influence over the associated microbiome. Depicting the algal associated microbial diversity and function is crucial to improve our comprehension of processes important for the holobiont resilience under environmental changes.

## Methods

### Sample collection and experimental design

Rhodoliths were collected at a depth of 10–12 m at Avalon harbor (33.3477^o^N, 118.3246^o^W), Catalina Island, California, USA in April 2014. The morphologic features of Catalina Island rhodoliths are consistent with the species *Sporolithon australe* [59]. Rhodoliths were collected by SCUBA divers and transferred to a laboratory holding tank at USC Marine Science Center (Catalina Island, CA, USA) filled with cycling surrounding seawater at the same natural conditions (temperature and nutrients) for a period of two days. Specimens were then transferred in dark coolers to the San Diego State University Coastal Marine Institute Laboratory (CMIL) (San Diego, CA). Once at the CMIL, rhodoliths were sorted to remove epibionts and associated fauna and then acclimated in incubation mesocosms with recirculating seawater collected off Scripps pier (San Diego, CA). Due to the constraints of collecting representative amounts of dead thalli in the field, the dead rhodoliths (calcareous skeleton) were obtained by sacrificing a subset of the live rhodoliths by drying them at 90 °C for 96 h and then bleaching them in sodium hypochlorite solution for 2 h. This treatment aimed to remove any algal live tissue and native associated microbes. The dead rhodoliths (calcareous skeleton) followed a 10-day period of acclimation on the control experimental conditions prior to the beginning of the experiment, in order to provide an initial colonization period for biofilm formation on the clean dead rhodolith surface.

The experimental setup was performed, with few modifications, following Brown [60]. Briefly, the experimental design incorporated two different algae treatments (live rhodoliths and calcareous skeleton from dead rhodoliths) and *p*CO_2_ at nearly present (ambient air) or predicted future (high *p*CO_2_) conditions, in duplicate mesocosms. Approximately 500 g of live and dead rhodoliths were placed in separated 18.5 L acrylic mesocosm tanks: tanks 1A and 1B for live rhodolith, ambient air treatment; tanks 2A and 2B for dead rhodolith, high CO_2_ treatment; tanks 3A and 3B for live rhodolith, high CO_2_ treatment; tanks 4A and 4B for dead rhodolith, ambient air treatment (*n* = 8 total). Every two mesocosms were connected to one of four 400 L seawater holding tanks (*n* = 2 mesocosms per large holding tank) in a recirculating system. The large holding tanks were used to maintain a stable *p*CO_2_ levels during the experiment, which is not possible to achieve by injecting the CO_2_ into the smaller mesocosm [35, 54]. It must be noted that this logistic constraint associated with our seawater system required that the duplicate mesocosms for each treatment were cultured receiving the water supply of a single large holding tank (i.e., duplicated mesocosms and seawater holding tanks could not be independently replicated). Despite the well-known problems of interpreting data based on pseudoreplicated technical samples [61], we point out that this is not uncommon in climate change mesocosm studies [62, 63] and relevant measurements in our study assessed the biological replicates of individual holobionts.

### Monitoring of the carbonate system

Seawater *p*CO_2_ levels within each large holding tank were adjusted to either ambient air (ca 500 μatm) by bubbling ambient air (the average yearly *p*CO_2_ values observed within the Southern California coast [60]); or high *p*CO_2_ (ca 1,500 μatm) by bubbling a certified CO_2_ mixture (1,485 ppm *p*CO_2_, Praxair San Diego) into the large holding tanks. The predicted increases in seawater *p*CO_2_ (Intergovernmental Panel on Climate Change 2007) are based on models for the open-ocean where values are expected to track atmospheric concentrations, and therefore they may not apply to coastal ecosystems where these values are much more dynamic (e.g., [64]). Along the southern coast of California, USA, pH values can vary by 0.2–0.4 pH units over the course of a single day [64], naturally exposing organisms to lower pH values compared to organisms in less variable environments like the open ocean. To ensure *p*CO_2_ levels remained at the desired levels, seawater samples were taken daily from the large holding tanks and analyzed using potentiometric titration to determine total alkalinity (A_T_) and total inorganic carbon (C_I_) [65]. The titration system consisted of a Metrohm 765 Dosimat titrator and Orion 920A pH meter. C_I_ and A_T_ could then be entered into the CO2Sys software (http://cdiac.ornl.gov/ftp/co2sys/) to calculate pH and *p*CO_2_ at a constant temperature and salinity [66] (Additional file 5).

Temperature in the holding tanks was maintained at 15 °C and controlled using aquarium chillers. Light above the mesocosms was provided by full spectrum fluorescent bulbs set on a 12:12 cycle, with light levels set at ~ 250–300 photons (μmol m^− 2^ s^− 1^), according to previous descriptions of the conditions within Catalina Island rhodoliths beds [67].

### Rhodolith physiology

To determine the effects of elevated *p*CO_2_ on rhodolith holobiont photosynthesis, photosynthetic carbon uptake was measured at the end of the 40-day experiment. Live algae from two *p*CO_2_ levels (500 μatm and 1500 μatm) treatments were measured separately over four consecutive days (for both duplicates mesocosms tanks A and B). On each day, 500 mL BOD bottles were filled with seawater taken from the mesocosm being measured, sealed and stored in the dark within a temperature-controlled room, set to 15 °C. A separate water sample was taken from the large holding tanks to determine starting C_I_ for each treatment. Concurrently, three separate rhodolith specimens (~ 0.7 g wet weight) were taken from the mesocosm being evaluated. Each rhodolith specimen was patted dry, weighed and placed into a BOD bottle. These bottles were then placed upright in separate water baths within the temperature-controlled room and photosynthetic carbon uptake by the algae was measured under six irradiances (0, 25, 50, 100, 250, and 750 photons (μmols m^− 2^ s^− 1^)) for one hour each. Light was provided by full-spectrum compact fluorescent bulbs hung directly above the bottles and light levels were adjusted either by moving the bulb closer to the bottles, or through window tinting of different shading properties until reaching the desired irradiance as measured with a photometer. To reduce the formation of boundary layers, magnetic stirring bars were placed into each bottle. After one hour, the rhodoliths were removed and the C_I_ within the bottles was measured using potentiometric titration as described above. Carbon uptake was calculated as the difference between C_I_ at the end of one hour versus the treatment’s starting C_I_ and standardized to the weight of the rhodolith. The rhodolith specimens were transferred to new bottles and the irradiance was increased to the next level. For each mesocosm, the carbon uptake at each light level was plotted and fit as in Platt [68]. From these best-fit lines, the maximum rate of photosynthesis (P_max_) and the photosynthetic efficiency (α) of each treatment were calculated.

Calcium carbonate loss was calculated on live rhodoliths only, by comparing the proportion of live tissue present on a subset of rhodoliths at the start of the experiment (day 1) and again at the end (day 40), according to Price [69]. To conduct the comparison, approximately, 0.3 g of rhodolith (the approximated weight of 1 cm^2^ diameter rhodolith individual) sampled at the beginning and end of the experiment, under both ambient air and high CO_2_ conditions, were weighed in triplicate and decalcified using formic acid. The rhodoliths were oven dried and weighed at the start and end of the decalcifying process, the mass difference after the removal of calcium carbonate skeleton was considered the proportion of flesh tissue in each sample. The amount of inorganic carbon (calcareous skeleton) was calculated as the total weight minus weight of organic material. The percentage (%) of inorganic material was calculated as (weight of inorganic carbon/total weight × 100). Student’s T-test was used to compare the proportion of tissue in the rhodoliths at the start (day 1) and end (day 40) of the experiment.

### DNA extraction and construction of metagenomic libraries

At day 0, day1, and day 7 of experiment two 4 L seawater samples from each large holding tank (*n* = 4) were collected and filtered using Sterivex filters (0.22 μm), the holding tanks are independent of each other. Microbial DNA extraction was performed using a modified column purification protocol (Nucleospin Tissue, Macherey-Nagel, Dueren, Germany) with proteinase K (final concentration 0.2 μg/mL), followed by incubation at 55 °C with gentle agitation overnight. By the end of the experiment (day 40), approximately 60–80 L of water from each large holding tank were collected and processed through tangential flow filters (TFFs). The filtrate was concentrated to around 500 ml on a 100 kDa TFF, and then passed through 0.22 μm Sterivex filters (Millipore, Inc) using a 60 ml syringe.

Rhodolith gDNA extraction was performed using multiple whole rhodolith individuals (~ 1 cm^2^ diameter) from each duplicate mesocosm (A and B) under both *p*CO_2_ levels (ambient air and high CO_2_) for the live rhodolith treatment; and one mesocosm (A) of both *p*CO_2_ levels (ambient air and high CO_2_) for the dead rhodolith treatment. The samples were collected at day 1 and day 40, i.e., the beginning and end of the experiment. All rhodolith samples were macerated with liquid nitrogen using sterile technique. The resulting slurry was processed using CTAB buffer with 100 mM of EDTA and the PowerSoil® DNA Isolation Kit (MoBio Laboratories, Carlsbad, CA, USA). A purification column was used to recover high-molecular-weight DNA as described by [70].

Water metagenomic libraries were prepared using a XT Nextera DNA sample Preparation Kit (Illumina) (*n* = 16). Rhodolith holobiont libraries were prepared using Accel-NGS 2S DNA Library Kit for Illumina platform (Swift Biosciences) (*n* = 12). The two library preparation kits were used because the NGS 2S kit provides better recovery from samples with low DNA concentrations. Variations between the two kits occur at the extreme GC range only (application note from Swift Biosciences). All metagenomic libraries were sequenced on an Illumina MiSeq (MiSeq Reagent Kit v3) at San Diego State University.

### Metagenomic sequence analysis

Metagenomic sequence reads were quality filtered using the PReprocessing and INformation of Sequences tool, PRINSEQ [71] to remove artificial replicate [72], while low quality sequence reads were trimmed to contain less than 5 bases of a Q-score ≤ 15 [73]. The quality filtered metagenomes were submitted to the MG-RAST 3.1 server (Metagenomics-Rapid Annotation Using Subsystems Technology) [41]. Post quality-control (QC) sequences were annotated using the (SEED) Subsystems Technology for functional classification [74] and the GenBank (M5 non-redundant) database for taxonomic analyses, similar to previous metagenomic analysis [28, 35, 75–77]. All BLAST queries were performed with a maximum expected cutoff value of 10^− 5^, 60% of minimum sequence identity and at least 15 bp alignment length. The MG-RAST server is robust in offering accurate taxonomic assignment to algal holobiont metagenomes comprised by distinguishable contributions between Eukaryotic and Prokaryotic domains, as exemplified in the metagenomic signature of Turfs assemblages, complex environmental algal samples, by Walter and collaborators [32].

Statistical analysis was performed using the Statistical Analysis of Metagenomic Profiles (STAMP v.2.0.0) software [78]. For multiple group comparisons, seawater and rhodolith metagenomes were compared by treatments: algae condition (live versus dead rhodolith) and *p*CO_2_ level. The multiple groups were compared by analysis of variance using a Tukey-Kramer post hoc test, and a Bonferroni multiple test correction. Comparisons between two groups were performed with the two-sided Welch’s t-test with 95% confidence intervals calculated by inverting the Welch’s test and by using the Bonferroni multiple test correction. In all these cases, *p*-values < 0.05 were considered statistically significant. A principal component analysis (PCA) was conducted using the R statistical software package to compare the functional grouping based on the SEED subsystems (level 1) [74] of all 28 metagenomes generated in the study.

Multidimensional scaling (MDS) plots were used with the annotated metagenome data to visualize the similarities between the rhodolith microbiome and the microbial profile of seawater in terms of taxonomy structure. Taxonomy structure was determined by comparing the relative abundances of 15 higher-rank bacterial classes, eukaryotic classes and archaeal families, in separate analysis. Groupings depicted by the MDS were based on the Bray**-**Curtis similarities, using either the statistical package PRIMER 6 (Plymouth Routines In Multivariate Ecological Research) or R. Comparisons over time of percentage changes in the relative abundance of taxa/functions in any treatment were calculated as (Δf / f_d1_) × 100, where Δf = f_d40_ – f_d1_ (relative abundance in day 40 – relative abundance in day 1).

Changes in bacterial communities between day 1 and day 40 of the experiment were calculated based on Bray-Curtis similarities among each of the rhodolith thallus treatments (i.e. live and dead rhodoliths), and each of the two seawater locations (i.e. water column over the live and dead rhodoliths). Prior to further testing, all Bray-Curtis similarity data were examined for normality by visual examination of probability plots, with the data plotted against a normal distribution [79], and for and equal variances using F-tests. All data were found to satisfy the assumptions of parametric statistics. Following this, differences in Bray Curtis similarities between locations (seawater column versus rhodolith thalli) and treatments (live versus dead) were then examined using a 2-way ANOVA, followed by Tukey’s post hoc tests on the significant location x treatment interaction (see Results).

All metagenomes analyzed here are available through MG-RAST under the project “Rhodolith_Calcareous Algae under Ocean Acidification” following the accession numbers as listed in Additional file 1.

## Additional files

Additional file 1: General features of metagenomes. (XLS 45 kb)

Additional file 2; Taxonomic similarities of Eukarya class (a) and Archaea family (b) in live rhodoliths, dead rhodolith biofilms and seawater metagenomes during the experiment. (EPS 3233 kb)

Additional file 3: Relative abundance of bacterial families in Rhodolith (live and dead) metagenomes (1% > hits contribution). (EPS 4059 kb)

Additional file 4: Functional profile. (a) Rhodolith metabolic profile for both live algae (left column) and dead algae (right column) under ambient air and high CO_2_ in days 1 and 40. (b) Metabolic profile of seawater tanks for both live treatment (left column) and dead treatment (right column) under ambient air and high CO_2_ in days 1 and 40. (EPS 7580 kb)

Additional file 5: Average water chemistry in treatment tanks. (XLS 28 kb)

## Abbreviations

A_T_: Total alkalinity
CCA: Crustose coralline algae
C_I_: Inorganic carbon
MDS: Multidimensional scaling
MG-RAST: Metagenomics-Rapid Annotation Using Subsystems Technology
OA: Ocean acidification
PCA: Principal component analysis
*p*CO_2_: Partial pressure of CO_2_
P_max_: Maximum potential photosynthetic
PRINSEQ: PReprocessing and INformation of Sequences
STAMP: Statistical Analysis of Metagenomic Profiles
T1A: Tank 1 – replicate A, live rhodolith under ambient air treatment
T1B: Tank 1 – replicate B, live rhodolith under ambient air treatment
T2A: Tank 2 – replicate A, dead rhodolith under high CO_2_ treatment
T2B: Tank 2 – replicate B, dead rhodolith under high CO_2_ treatment
T3A: Tank – replicate A, live rhodolith under high CO_2_ treatment
T3B: Tank – replicate B, live rhodolith under high CO_2_ treatment
T4A: Tank 4 – replicate A, dead rhodolith under ambient air treatment
